# Comprehensive pre- and in-hospital near-infrared-spectroscopy (NIRS) monitoring after return of spontaneous circulation predicts neurological outcome following out-of-hospital cardiac arrest: a prospective observational study and literature review

**DOI:** 10.3389/fmed.2025.1590908

**Published:** 2025-08-15

**Authors:** Sebastian Schnaubelt, Andrea Kornfehl, Felix Eibensteiner, Christoph Schriefl, Florian B. Mayr, Patrick Aigner, Mathias Gatterbauer, Michael Girsa, Daniel Grassmann, Andreas Zajicek, Alexander Spiel, Wolfgang Schreiber, Michael Holzer, Heidrun Losert, Mario Krammel, Thomas Uray

**Affiliations:** ^1^Department of Emergency Medicine, Medical University of Vienna, Vienna, Austria; ^2^PULS – Austrian Cardiac Arrest Awareness Association, Vienna, Austria; ^3^Emergency Medical Service Vienna, Vienna, Austria; ^4^Department of Critical Care Medicine, VA Pittsburgh Healthcare System, and CRISMA Center, University of Pittsburgh, Pittsburgh, PA, United States; ^5^Department of Emergency Medicine, Clinic Ottakring, Vienna Healthcare Group, Vienna, Austria

**Keywords:** cerebral oxygenation, near-infrared spectroscopy, NIRS, cardiac arrest, cardiopulmonary resuscitation, CPR, prognostication

## Abstract

**Background:**

An increase in regional cerebral oxygen saturation (rSO2) levels during advanced life support in patients with out-of-hospital cardiac arrest (OHCA) is associated with return of spontaneous circulation (ROSC) and can predict neurological outcome. Data from the post-ROSC phase are scarce but may predict clinical outcomes as well.

**Methods:**

For this prospective observational study, we measured rSO2 via near-infrared spectroscopy (NIRS) in patients after ROSC following OHCA in both the pre- and in-hospital setting for up to 72 h. Patients were followed up for their post-ROSC treatment and outcomes. NIRS values were then compared between patients with favorable and non-favorable neurological outcomes, and cutoff values were assessed via receiver operating characteristic (ROC) and Classification and Regression Trees (CART) analyses. In addition, a narrative review on the topic was performed.

**Results:**

Of the 27 included patients, 37% survived hospital discharge, and 26% showed favorable neurological outcomes (CPC 1 or 2). RSO2 was significantly higher in individuals with CPC 1/2 (67 [60–69] % vs. 59 [50–70] %; *p* = 0.049). This was even more pronounced for initial (= a mean of the first 5 min) NIRS values (70 [65–77] % vs. 57 [49–68] %; *p* = 0.025) and NIRS values rising in the first 10 min (43% vs. 5% of patients; *p* = 0.042). A ROC analysis for initial rSO2 showed a significant discriminatory power to predict CPC 1/2 (AUC = 0.789, *p* = 0.025), and both ROC and CART analyses suggested an optimal cutoff of approximately 62% rSO2.

**Conclusion:**

We identified a potential RSO2 cutoff measured via NIRS in the post-ROSC phase after OHCA to predict favorable neurological outcomes. Initial values and rising trends may be more useful for prognostication than prolonged ICU measurements. These findings are consistent with previous literature and should prompt both larger clinical trials and consideration of this technology by resuscitation societies.

## 1 Introduction

Near-infrared spectroscopy (NIRS) is used to assess and monitor regional cerebral oxygenation (rSO2) in various settings, including anesthesia, perioperative medicine, and intensive care (1, 2). Near-infrared light emitted from optodes placed on the forehead penetrates underlying tissues and is then reflected back, with varying degrees of absorption depending on the oxygenated status of the molecules. A percentage of tissue oxygen saturation is then calculated. Thus, a lower rSO2 value can serve as a surrogate for reduced frontal cerebral oxygenation and cerebral perfusion, which is otherwise not typically monitored in critical care (1–3). Optimizing cerebral (hypo-)perfusion is a cornerstone of cardiopulmonary resuscitation (CPR) for in- or out-of-hospital cardiac arrest (IHCA / OHCA) treated by basic and advanced life support (BLS / ALS), as prolonged cerebral ischemia leads to dismal neurological outcomes, even if return of spontaneous circulation (ROSC) may have been achieved (4). Therefore, following in the footsteps of end-tidal carbon dioxide (etCO2) (5), rSO2 has been evaluated as a non-invasive prognostication tool during ALS and post-resuscitation care. Higher rSO2 seems to be associated with favorable outcomes during and after CPR, but there is a lot of variation, no consensus on which values predict favorable or unfavorable outcomes, and no clear cutoffs (4, 6–9). Accordingly, current guidelines do not recommend the routine use of NIRS in resuscitation care, but on the other hand, they recognize its potential for future use (5, 10). Furthermore, the most up-to-date consensus on respective science by the International Liaison Committee on Resuscitation (ILCOR) states there are insufficient data for a recommendation for or against the routine use of NIRS (11). Interestingly, an AHA statement on neuroprognostication in comatose cardiac arrest survivors does not mention NIRS (12), whereas a more recent joint statement by the AHA and Neurocritical Care Society on critical care management of patients after cardiac arrest states that NIRS could play a role in post-resuscitation care, and calls for further research (13).

We therefore aimed to investigate: (1) the feasibility of using NIRS during post-resuscitation care, starting directly after ROSC, regardless of whether in pre- or in-hospital setting; and (2) its prognostic value when measured for the subsequent 72 h.

## 2 Methods

### 2.1 Study setting and population

For this prospective observational study, patients who received ROSC after OHCA in Vienna, Austria, were included between November 2016 and September 2018. Further inclusion criteria were: ≥ 18 years old, OHCA in the metropolitan area of Vienna, treated by the Emergency Medical Service (EMS) Vienna, and then treated at the Emergency Department (ED) and/or an Intensive Care Unit (ICU) of the Medical University of Vienna. Exclusion criteria were traumatic cardiac arrest, known or suspected pregnancy, and a decision of the team on site that a NIRS measurement was not possible (e.g., due to logistic or space constraints). Patient selection followed a real-world pragmatic approach: all patients seen by the so-called Field Supervisors (FISU; paramedics specially trained in crew resource management and quality management deployed to high-priority missions) of EMS Vienna (14) during the observational period were screened for eligibility to measure NIRS. RSO2 was measured using a NONIN SenSmart^®^ X-100 (Nonin Medical Inc., Plymouth, United States) device (15). One optode was placed on the right forehead of each patient, and one on the left forehead of each patient, and data points were saved every 4 s. The device was lightweight and portable. Inclusion was possible either in the pre- or the in-hospital setting (at the OHCA scene, the ambulance, or the ED—depending on where ROSC occurred) and was continued for up to 72 h post-ROSC (at the intensive care unit—depending on the duration of survival). Gaps between ROSC and the start of NIRS were excluded from any trend analysis. The same device was used for each patient throughout their measurement period, without planned interruptions. The included patients were all treated following the EMS’s and the hospital’s standard operating procedure for post-cardiac arrest care. Any ongoing CPR during transportation was delivered via a mechanical chest compression device. To achieve a comprehensive measurement strategy and maximize compliance, the NIRS device was handled by dedicated personnel, as described previously. In the observational period, this was still a pilot project, which resulted in the presented low patient numbers (14).

Ethical approval for this study (no 1265/2015) was acquired from the Ethical Committee of the Medical University of Vienna, Austria, and informed consent was waived. The study protocol complies with the Declaration of Helsinki.

### 2.2 Narrative review

To provide a summary of the existing literature on NIRS in post-resuscitation care, we performed a narrative review. Medline/PubMed and Embase were searched using the terms “Near-infrared spectroscopy,” “NIRS,” “cerebral oxygen,” “cerebral oxygenation,” and “rSO2,” in combination with “cardiac arrest,” “resuscitation,” “life support,” “CPR,” “return of spontaneous circulation,” “ROSC,” “intensive care,” “critical care,” and “emergency medicine.” Relevant abstracts were screened, and respective information was extracted from the full-texts.

### 2.3 Statistical analyses

Data were assessed for normal distribution using a Kolmogorov–Smirnov test. Continuous data are presented as medians and the respective interquartile ranges (IQRs) and were compared among subgroups using the Mann–Whitney *U*-test. Categorical data are presented as counts and percentages and are compared using the χ^2^-square test where appropriate. Receiver operating characteristic (ROC) analyses were utilized to assess the discriminatory power of specific cutoff levels and thresholds as potential indicators for clinical decision-making. A classification and regression tree (CART) analysis was conducted to elucidate the importance of NIRS values in a predictive model and to establish a cutoff value. Statistical significance was defined by two-tailed *p*-values of <0.05. Data analysis was performed using SPSS 22.0 (IBM, United States).

## 3 Results

We included 27 patients with OHCA who had been treated with standard ALS, reached sustained ROSC, and then received NIRS measurements. None regained consciousness immediately after ROSC. No extracorporeal CPR (eCPR) patients were included. In 10 patients, the NIRS measurement was started in the pre-hospital setting, and in 17 patients in the in-hospital setting after ED admission (ROSC after ED admission). There were no re-arrests during the observational period. Ten patients (37% of all included) survived to hospital discharge, and seven of them (26% of all included or 70% of the survivors to hospital discharge) had favorable neurological outcomes (CPC 1 or 2; whereas six patients reached CPC 1, one patient CPC 2, two patients CPC 3, and one patient CPC 4). Modified Rankin Scale (mRS) at hospital discharge was excellent (1.5 ± 0.5) in those reaching a CPC of 1 or 2. Further patient characteristics are shown in Table 1, and Supplementary Table S1 shows the comparison between pre-hospital and in-hospital measurements.

**Table 1:** Basic demographics of the study population and respective CPR and NIRS characteristics.

Basic-, CPR-, and NIRS characteristics				
	Total (*n* = 27)	Favorable neurological outcome at hospital discharge (CPC 1/2; *n* = 7)	No favorable outcome (CPC 3–5; *n* = 20)	*p*-value
Age, years (95% CI)	59 (53–73)	55 (32–60)	63 (54–78)	0.108
Male, *n* (%)	20 (74)	5 (71)	15 (75)	0.989
Witnessed CA, *n* (%)	22 (82)	7 (100)	15 (75)	0.283
Bystander-CPR, *n* (%)	21 (78)	7 (100)	14 (70)	0.155
Layperson AED use, *n* (%)	2 (8)	1 (14)	1 (5)	0.459
Cardiac origin of CA, *n* (%)	18 (67)	7 (100)	11 (55)	**0.050**
Shockable initial rhythm, *n* (%)	14 (52)	6 (86)	8 (40)	0.081
Total shocks, *n* (95% CI)	1 (0–3)	2 (1–3)	0 (0–4)	0.384
Airway procedure				
Primary endotracheal intubation, *n* (%)	19 (70)	5 (71)	14 (70)	0.997
Primary larynx tube, *n* (%)	4 (15)	1 (14)	3 (15)	0.893
Primary bag-valve-mask ventilation only, *n* (%)	4 (15)	2 (29)	2 (10)	0.995
Epinephrine – cumulative dose during CPR, mg (95% CI)	3 (1–6)	1 (0–2)	4 (2–6)	**0.013**
Amiodarone given during CPR, *n* (%)	6 (22)	1 (14)	5 (25)	0.456
No- and low-flow time (CA to ROSC), hours (95% CI)	0.35 (0.23–0.52)	0.27 (0.23–0.40)	0.38 (0.22–0.75)	0.258
Delay from ROSC to start of NIRS measurement, hours (95% CI)	0.94 (0.54–1.32)	1.03 (0.60–1.59)	0.88 (0.50–1.11)	0.684
Duration of NIRS measurement, hours (95% CI)	17.88 (0.77–72.03)	66.13 (36.01–77.72)	4.09 (0.42–67.1)	**0.013**
Overall NIRS values, %rSO2 (95% CI)	64 (50–69)	67 (60–69)	59 (50–70)	**0.049**
Initial NIRS values post-ROSC, %rSO2 (95% CI)	65 (50–73; min. 30, max. 84)	70 (65–77; min. 63, max. 81)	57 (49–68; min. 33, max. 82)	**0.025**
NIRS values increasing in the first 10 min post-ROSC, *n* (%)	4 (15)	3 (43)	1 (5)	**0.042**
NIRS values at 24 h post-ROSC, %rSO2 (95% CI)	74 (70–76; min. 55, max. 81)	67 (63–77; min. 60, max. 79)	68 (57–79; min. 48, max. 83)	0.943
NIRS values at 48 h post-ROSC, %rSO2 (95% CI)	70 (67–73; min. 66, max. 75)	72 (71–77; min. 71, max. 77)	72 (64–73; min. 57, max. 74)	0.523
NIRS values at 72 h post-ROSC, %rSO2 (95% CI)	70 (63–80; min. 62, max. 81)	70 (66–74; min. 66, max. 74)	70 (63–72; min. 63, max. 76)	0.989

Continuous data are presented with their means and standard deviation (SD) and were compared using the Mann–Whitney *U*-test. Discrete data are presented as counts and percentages and were compared using the chi-square test. CPR = cardiopulmonary resuscitation; NIRS = near-infrared spectroscopy; CPC = cerebral performance category; CA = cardiac arrest; AED = automated external defibrillator; ROSC = return of spontaneous circulation. Bold *p*-values depict statistically significant findings.

The overall length of hospital stay (until death or discharge) was 4 (1–17) days. In the subgroup of patients who survived to hospital discharge, it was 20 (14–37) days. Standard post-resuscitation care was applied to all patients, and, if applicable, reversible causes of cardiac arrest were treated (e.g., PCI for a presumed cardiac origin was performed in 67% of cases). All patients received targeted temperature management (active cooling, 32–34°C for 24 h) and were mechanically ventilated (pressure-controlled or pressure-regulated volume controlled). See Table 2 for more information about post-ROSC care, including catecholamine support and prognostication, and Supplementary Table S2 for details on ventilation settings and blood gas analyses.

**Table 2:** Details on post-ROSC care.

Post-ROSC care				
	Total (*n* = 27)	Favorable neurological outcome at hospital discharge (CPC 1/2; *n* = 7)	No favorable outcome (CPC 3–5; *n* = 20)	*p*-value
Length of hospital stay (until death or discharge), days (95% CI)	4 (1–17)	19 (15–23)	1 (1–8)	**0.003**
Invasive ventilation				
Initial, *n* (%)	27 (100)	7 (100)	20 (100)	N.A.
24 h, *n* (%)	15 (56)	7 (100)	8 (40)	0.748
48 h, *n* (%)	12 (44)	4 (57)	8 (40)	0.077
72 h, *n* (%)	10 (37)	3 (43)	7 (35)	0.070
Catecholamine support				
Initial				
Norepinephrine, *n* (%)	15 (56)	6 (86)	9 (45)	0.091
Dobutamine, *n* (%)	3 (11)	0	3 (15)	0.545
24 h				
Norepinephrine, *n* (%)	13 (48)	6 (86)	7 (35)	0.821
Dobutamine, *n* (%)	2 (7)	1 (14)	1 (5)	0.974
48 h				
Norepinephrine, *n* (%)	10 (37)	4 (57)	6 (30)	0.608
Dobutamine, *n* (%)	2 (7)	1 (14)	1 (5)	0.974
72 h				
Norepinephrine, *n* (%)	6 (22)	2 (29)	4 (20)	0.592
Dobutamine, *n* (%)	0	0	0	N.A.
Prognostication				
Cerebral edema seen on CT within 72 h, *n* (%)	4 (15)	0	4 (20)	**0.048**
NSE				
24 h, μg/L (95% CI)	29 (20–44)	29 (20–44)	37 (18–84)	**0.050**
48 h, μg/L (95% CI)	20 (13–44)	20 (13–44)	21 (12–154)	0.121
72 h, μg/L (95% CI)	12 (11–25)	12 (11–25)	15 (11–80)	0.800

Continuous data are presented with their means and standard deviation (SD) and were compared using the Mann–Whitney *U*-test. Discrete data are presented as counts and percentages and were compared using the chi-square test. CT = computed tomography; NSE = neuron-specific enolase. Bold *p*-values depict statistically significant findings.

NIRS was measured up to 72 h post-ROSC (see Supplementary Figure 1 for details of the measurement duration). The median duration of NIRS was 17.88 (0.77–72.03) h (all patients). Cerebral oxygenation was significantly higher in individuals reaching a favorable neurological outcome (67 [60–69] % vs. 59 (50–70) %; *p* = 0.049). Between patients with a CPC of 3/4 and 5, there was no significant difference in rSO2 (Supplementary Table S3). When evaluating various points of time during the measurement period, this difference was even stronger for initial (= a mean of the first 5 min) NIRS values (70 [65–77] % for CPC 1/2 vs. 57 (49–68) % for CPC 3–5; *p* = 0.025) and NIRS values rising within the first 10 min (43% of patients for CPC 1/2 vs. 5% of patients for CPC 3–5; *p* = 0.042). No significant difference was detectable beyond 24 h. See Table 1 for an overview of the NIRS values and respective comparisons.

**Figure 1:**
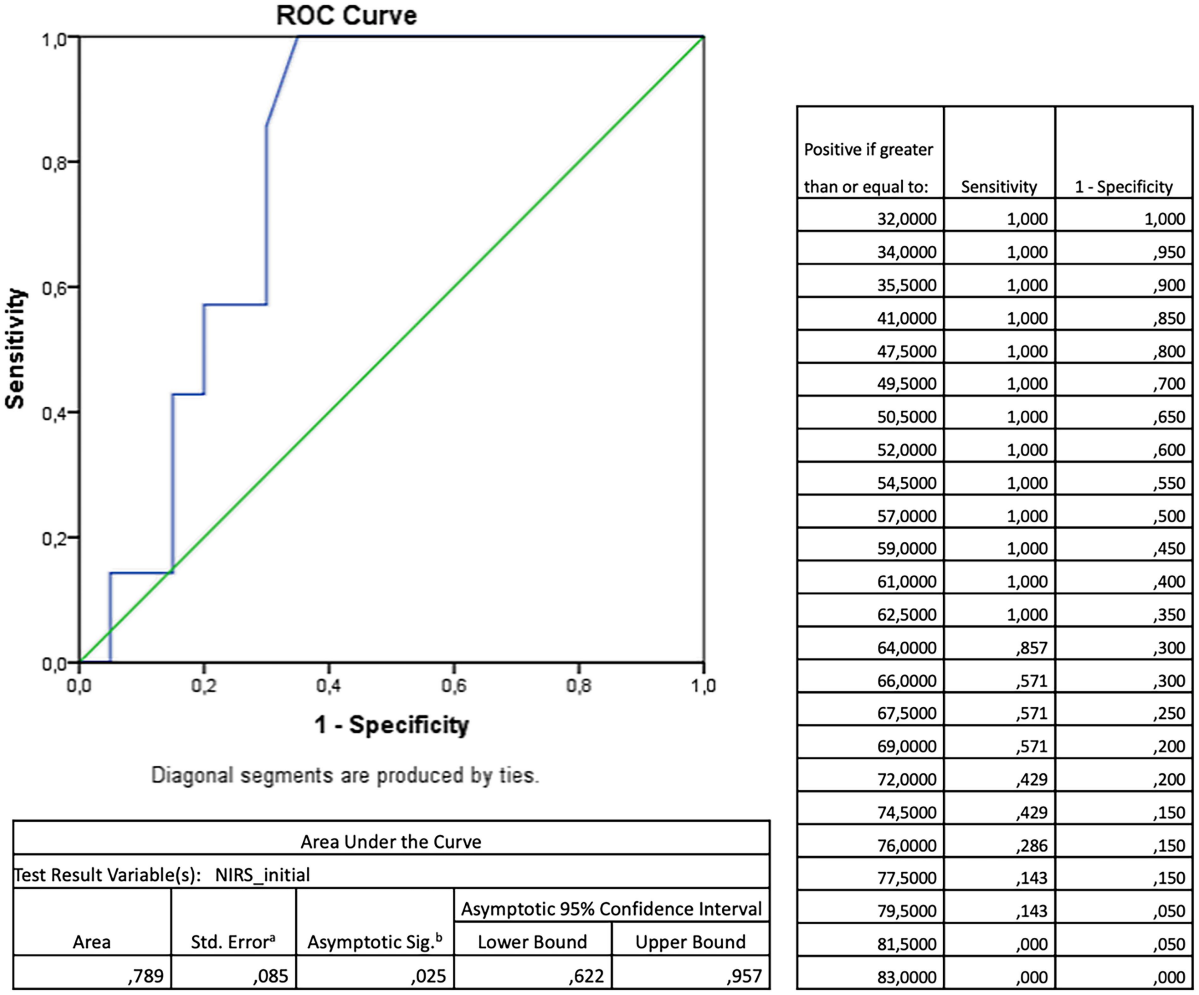
Receiver operating characteristic (ROC)—analysis for the prognostic value of the initial (= a mean of the first five minutes) cerebral oxygenation measured by near-infrared spectroscopy (NIRS) towards favourable neurological outcome measured by cerebral performance category (CPC) 1 or 2 after return of spontaneous circulation after out-of-hospital cardiac arrest.

**Figure 2:**
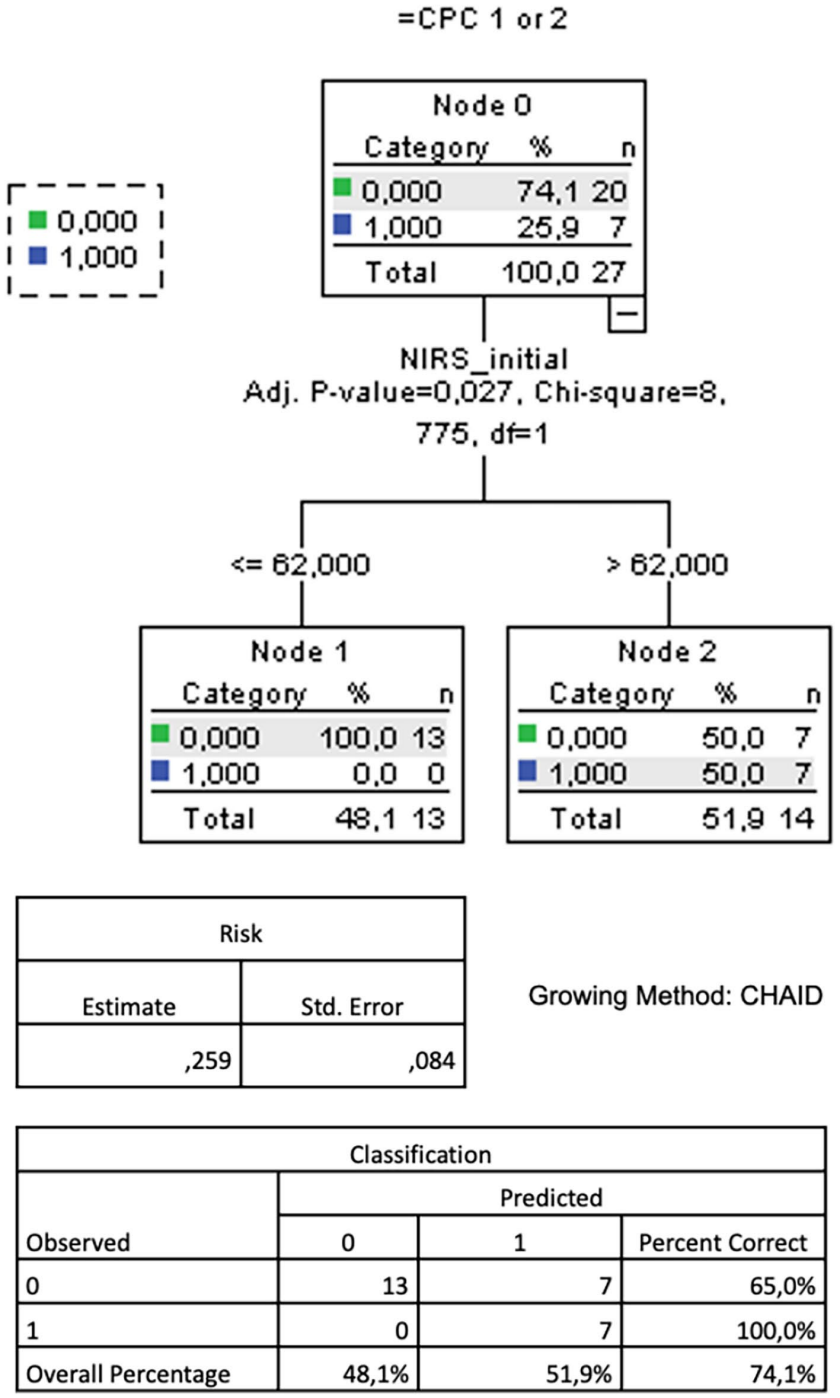
CART analysis (growing method CHAID) for the prognostic value of the initial (= a mean of the first five minutes) cerebral oxygenation measured by near-infrared spectroscopy (NIRS) for favourable neurological outcome measured by cerebral performance category (CPC) 1 or 2 after return of spontaneous circulation after out-of-hospital cardiac arrest. CART = classification and regression tree. CHAID = Chi-square Automated Interaction Detection.

A ROC analysis of the rSO2 values during the whole measurement period to predict a favorable neurological outcome yielded no significant result. However, an additional ROC analysis of the initial NIRS values (Figure 1) showed their significant discriminatory power to predict a favorable neurological outcome (AUC = 0.789, *p* = 0.025). An rSO2 cutoff of 62.5% predicted a CPC of 1 or 2 with a sensitivity of 100% and a specificity of 65%. Accordingly, a CART analysis for the prognostic value of the initial rSO2 resulted in an optimal cutoff value of 62% to be highly predictive of a favorable neurological outcome, with an overall correct classification rate of 74.1% (Figure 2).

## 4 Discussion and literature review

Our three key findings were that (1) an early NIRS measurement after ROSC is feasible, regardless of whether in the pre- or in-hospital setting, (2) rS02 levels after ROSC can predict favorable neurological outcomes, and (3) early measurements and the trajectory of measurements appear to be more important than prolonged measurements over time.

As a complete novelty, we measured NIRS right from the time point when ROSC occurred, no matter whether still in the pre-hospital or already in the in-hospital setting. This approach was feasible.

Neither of the respective guiding documents by ILCOR, the AHA, or the ERC currently suggests the use of NIRS during or after cardiac arrest. However, it seems that until now, insufficient attention has been paid to the growing body of literature on it, as summative publications on post-resuscitation care often either include no or only singular NIRS studies, or studies on specific patient groups. Of note, most evidence is derived from or about rSO2 measurements during CPR and not a longer observational period after ROSC. This may have led to a somewhat incomplete picture of the technology’s prognostication possibilities (11, 16–22). Recently, a statement by the AHA and Neurocritical Care Society on critical care management of patients after cardiac arrest mentions NIRS to potentially contribute to a more individualized monitoring and treatment approach in the future, and suggests further research (13). While a body of evidence on NIRS during CPR is available, a consensus on cutoffs or target values has yet to be reached (4, 8, 18, 23–31). The situation is even more scarce and heterogeneous for post-ROSC measurements, and there are no robust data on a comprehensive and continuous rSO2 assessment during and after CPR (32).

### 4.1 Pre-hospital post-ROSC NIRS

A pattern of increasing rSO2 during CPR has been shown to be associated with ROSC and survival in the pre-hospital setting (8, 26, 27, 29, 30). Directly after ROSC, some patients suffer from repeated hemodynamic instability and/or re-arrests. Recognizing such states quickly is a challenge, especially in the pre-hospital setting, where usually no invasive blood pressure measurement is available, and (re-)assessing patients during transport might be challenging. NIRS, however, does not need pulsatile flow and can serve as additional monitoring to timely detect patient deterioration or re-arrest (3, 6, 29, 33–37), as well as guidance for interventions (38–40). Data on pre-hospital rSO2 measurements and their association with (neuro-)prognostication are even more scarce than in-hospital ones.

### 4.2 NIRS in post-resuscitation intensive care

ICU treatment after ROSC is increasingly focused on achieving favorable neurological outcomes and improving the quality of life of cardiac arrest survivors, rather than merely ensuring survival to hospital discharge. Post-cardiac arrest brain injury, the leading cause of mortality after CPR, can range from no or mild impairment to permanent disability or brainstem death, rendering neuromonitoring and prognostication increasingly important. A multimodal approach is applied, aiming at optimal personalized care (12, 21, 41–44). For instance, non-invasive neuromonitoring is increasingly being used in ICUs, either for primary brain injury or patients at risk of a secondary one. Apart from the features discussed above for the pre-hospital phase, here NIRS plays a part in a multimodal approach (13, 45), is non-invasive, does not require additional skills (46), and can identify cardiopulmonary instability (40) or monitor brain oxygen delivery and metabolism (47, 48). Furthermore, it seems more practical for widespread use than invasive techniques such as intracranial pressure monitoring (49). However, evidence on the clinical effects of rSO2 monitoring is still uncertain (50). There have been reports on rSO2 measurements during targeted temperature management after cardiac arrest, suggesting an rSO2 decrease after the onset of hypothermia and values normalizing again afterward (9, 51–56). In the complicated multi-organ post-cardiac arrest syndrome, cerebral autoregulation and blood pressure dynamics play an important part, opening a chance of preventing further cerebral injury, as well as promoting recovery (20). For instance, arterial pressure goals could be monitored via NIRS or special respective indices which could then serve as a surrogate for (impaired) cerebral autoregulation (7, 13, 49, 57–59).

### 4.3 Ranges and cutoffs

Post-ROSC rSO2 usually lies between 50 and 70%, with noticeable upwards trends the longer the measurement is continued (of note, there are no robust data on >72 h post-ROSC) (9, 48, 51, 56). This is in accordance with our results. However, single studies also report lower values of approximately 40% (55, 60). Despite reports of patients with low rSO2 values during CPR still reaching favorable outcomes (61), both available literature on post-ROSC NIRS and our study strongly suggest higher mean or median rSO2 values over a certain period of time (mostly up to 24, 48, or 72 h) to be associated with a favorable neurological outcome, albeit not always reaching statistical significance. Reported rSO2 values in the respective outcome subgroups are quite heterogeneous and range approximately 60–70% for CPC1/2 and 50–66% for CPC 3–5. However, there are overlaps that can make exact classifications into rSO2-groups difficult (6, 51–54, 56, 62–64). There have been reports of rSO2 differences between neurological outcome groups, “wearying off” or diminishing after a certain amount of time (48, 65)—an effect that we also observed in our cohort. Explanatory hypotheses for such dynamics include primary cerebral metabolic suppression after ROSC leading to a low cerebral oxygen extraction fraction, an oxygen supply/demand mismatch, cerebral ischemia due to instable hemodynamics, hypocapnia, small vessel caliber reduction, or impaired microvascular perfusion for lower-, and (reactive) hyperemia or hyperperfusion with increased cerebral blood flow for higher rSO2 values (45). Although prior reports have described a decrease in both cerebral metabolic rate of oxygen (CMRO₂) and oxygen extraction fraction (OEF) 24–72 h after cardiac arrest, suggesting a preserved global flow–metabolism coupling (42), our observation of described dynamics in rSO₂ may reflect regional and functional heterogeneities that are not captured by global metrics alone: First, cerebral blood flow (CBF) following cardiac arrest is often regionally heterogeneous, with imaging studies revealing coexisting zones of hyperemia, hypoperfusion, and no-flow, particularly in metabolically vulnerable regions such as watershed zones or the hippocampus (66). Second, impaired cerebrovascular autoregulation in the subacute phase may lead to inadequate regional perfusion in response to systemic changes (e.g., hypotension or vasopressor withdrawal), even when overall CMRO₂ is reduced (67). Third, NIRS-derived rSO₂ is susceptible to influences beyond pure flow–metabolism dynamics, including microvascular dysfunction, capillary flow heterogeneity, hemoglobin changes, or mitochondrial impairment limiting oxygen utilization despite adequate perfusion (67, 68). We could observe that rising rSO2 values in the first 10 min after ROSC led to a higher percentage of patients performing neurologically well—a more beneficial response to reperfusion and a faster cerebral recovery from hypoxia could be the reason, rendering NIRS a potential marker for individual cerebral reconstitution after ROSC.

The time points or durations of the measurements vary in the literature. While it seems to have become standard to not only measure at single points in time anymore (e.g., at ROSC and at hospital admission) and to rather use means or medians up until a certain time period after ROSC, the optimal approach is still unknown. It remains debatable whether either overall means/medians or dynamics (e.g., from a baseline or nadirs) are more beneficial (9, 32, 69). Rarely, only mortality, but not neurological function, has been assessed, showing a trend toward lower rSO2 values in non-survivors (48, 51, 70).

Naturally, a clear rSO2 cutoff to discriminate favorable vs. unfavorable outcome groups (or also other more secondary outcomes) would be a milestone in modern post-resuscitation care for both the pre-hospital (in addition to etCO2) and the in-hospital setting (in addition to invasive blood pressure monitoring). Accordingly, several receiver operating curve (ROC) analyses have in the past been conducted, with mixed results ranging from thresholds of 40% (area under the curve [AUC] 0.9, 81% sensitivity, 96% specificity) (64), 49% (AUC 0.8, 100% specificity, 45% sensitivity) (62), and 50% (AUC 0.8, 70% specificity, 86% sensitivity) (52), up to 55% (AUC 0.6, 52% specificity, 62% sensitivity) (9). When combined with NSE values, one study reported an optimal cutoff of 83% (AUC 0.9, 100% specificity, 76% sensitivity) (63). Other previous data showed no association of rSO2 and NSE (7). Since NSE should be assessed dynamically, correlating single NSE values with rSO2 levels has limited value.

Interestingly, we could not find a good overall rSO2 cutoff for a favorable neurological outcome in our cohort (whereas it was probably underpowered for this), but rather a threshold of 63% for the mean rSO2 over the first 5 min after ROSC (AUC = 0.8, 65% specificity, 100% sensitivity). With a high correct classification rate, this cutoff could be confirmed by CART analysis. This fits with the above-discussed finding of the beneficial effect of rSO2 values rising in the first 10 min after ROSC and underscores the hypothesis that values over such a threshold could mean a better post-ROSC individual adaptation. A comprehensive NIRS measurement in the pre- and in-hospital setting—wherever ROSC occurs—is a condition to “catch” these probably short-lived dynamics.

### 4.4 Knowledge gaps and outlook

Even though the described reports, including ours at hand, on NIRS in the post-ROSC phase have provided more insights into the topic, certain knowledge gaps persist:

- Is a unilateral NIRS measurement sufficient? Previous reports suggest so if there is no sign of unilateral pathology (71), and one instead of two optodes per patient would be more cost-efficient.
- What is the optimal mode of measurement/monitoring (means/medians, deltas, trends, nadirs, etc.)? Are there different optimal modes for different outcomes (e.g., initial values and dynamics after ROSC for neurological prognostication, such as in our study)?
- The relation between rSO2 and etCO2 should be further researched for its combined and supplemental prognostication potential, not only during but also after ROSC (72, 73).
- Do certain interventions (e.g., medication or ventilation details), as well as certain hemodynamic conditions (e.g., hemodynamically relevant dysrhythmia) in critical and intensive care, have an impact on rSO2? (23, 24)
- Is higher rSO2 necessarily directly associated with improved or preserved neurological function (also on a molecular level), or is it rather a surrogate for interventions, care, or inter-patient characteristics? (7)
- What is an rSO2 cutoff that is feasible for clinical use while providing sufficient sensitivity and specificity for a favorable neurological outcome?
- Is predicting an unfavorable neurological outcome clinically more relevant in post-ROSC ICU patients than a favorable outcome?
- Does a NIRS measurement in clinical use directly or indirectly impact more imminent clinical outcomes than neurological performance, such as, for instance, the duration and invasiveness of ventilation, episodes of hemodynamic deterioration, etc.?

In the future, not only should these knowledge gaps be addressed in large trials providing sensible endpoints with adequate power, but respective opinion-leading associations and societies should also acknowledge the growing body of literature on NIRS in resuscitation medicine as well. As one piece of the prognostication mosaic, NIRS does indeed show undeniable potential for the pre- and in-hospital setting. However, especially for its use by EMS crews, technical issues must be addressed first—ideally, NIRS would be integrated into already-existing ALS monitor-defibrillator devices.

Another interesting novel possibility to integrate such a multimodal approach into clinical practice is using artificial intelligence (74), which would probably profit from additional monitoring data without adding to the clinicians’ actual plate of work. It is imaginable that a linear approach (9) could then give real-time rSO2 trends combined with other prognostication factors.

## 5 Limitations

This pilot study is purely hypothesis-generating. One limitation is that we only report median values for the right and left optodes, which is, however, a common practice in previous literature. Another limitation is that the assessed data are not very new at the time of publication (this was due to organizational and resource reasons). Furthermore, due to resource limitations, we only assessed neurological outcome at hospital discharge and not at further timepoints afterward. We only used a convenience sample size, thus probably rendering our results underpowered and missing various effects (especially the patient group surviving >24 h was small). However, we did manage to obtain statistically significant results in some domains despite the low patient numbers in the subgroups, underscoring the reported effect. Moreover, patient selection was not controlled but followed a pragmatic real-life approach of FISUs, starting NIRS measurement whenever they were on scene and the patients were eligible. Furthermore, our subgroup with poor outcomes also showed a generally unfavorable prognostic profile (see Table 1), and even though these differences did not reach statistical significance, this was probably due to the low subgroup patient numbers. Of note, we did not have any information on rhythmology at the emergency department level or various interventions performed in the ICU, which potentially had impacted NIRS values during the observational period. We also did not have enough information on somatosensory evoked potentials, as they were either not assessed in our patients or the respective data were not available. Finally, we did not include eCPR patients, which could potentially impact our results. Our findings are of a hypothesis-generating nature only.

Regarding the narrative review section of this publication, the search strategy was designed without he involvement of an information specialist. Additionally, neither a scoping nor a systematic review was conducted, and no risk of bias assessments were performed, which limits the value of the summarized information.

## 6 Conclusion

We identified a potential RSO2 cutoff measured via NIRS in the post-ROSC phase after OHCA to predict a favorable neurological outcome. Initial values and rising dynamics could be more useful for prognostication than a prolonged measurement in the ICU. These findings fit into previous literature and should trigger larger respective trials on the one hand, and the technology being addressed by resuscitation societies on the other hand.

## Data Availability

The raw data supporting the conclusions of this article will be made available by the authors, without undue reservation.
