# Supplementary material for: Comprehensive pre- and in-hospital near-infrared-spectroscopy (NIRS) monitoring after return of spontaneous circulation predicts neurological outcome following out-of-hospital cardiac arrest: a prospective observational study and literature review
Source: Front Med (Lausanne). 2025 Aug 15;12:1590908. doi: 10.3389/fmed.2025.1590908 (PMC12394502; doi:10.3389/fmed.2025.1590908)
Supplement: Supplementary file 2 [file Table_1.DOCX]

|  | **Total (n=27)** | **Pre-hospital (n=10)** | **In-hospital (n=17)** |
| --- | --- | --- | --- |
| Age, years (95% CI) | 59 (53-73) | 68 (56-80) | 59 (47-66) |
| Male, n (%) | 20 (74) | 9 (90) | 12 (71) |
| Witnessed CA, n (%) | 22 (82) | 7 (70) | 17 (100) |
| Bystander-CPR, n (%) | 21 (78) | 8 (80) | 15 (89) |
| Layperson AED use, n (%) | 2 (8) | 1 (10) | 1 (6) |
| Cardiac origin of CA, n (%) | 18 (67) | 6 (60) | 13 (77) |
| Shockable initial rhythm, n (%) | 14 (52) | 3 (30) | 12 (71) |
| Total shocks, n (95% CI) | 1 (0-3) | 0 (0-3) | 2 (0-3) |
| Primary endotracheal intubation, n (%) | 19 (70) | 6 (60) | 16 (94) |
| Primary larynx tube, n (%) | 4 (15) | 2 (20) | 1 (6) |
| Primary bag-valve-mask ventilation only, n (%) | 4 (15) | 2 (20) | 0 |
| Epinephrine – cumulative dose during CPR, mg (95% CI) | 3 (1-6) | 6 (1-6) | 2 (0-4) |
| Amiodarone given during CPR, n (%) | 6 (22) | 2 (20) | 4 (24) |
| No- and low-flow time (CA to ROSC), hours (95% CI) | 0.35 (0.23-0.52) | 0.17 (0.13-0.30) | 0.33 (0.23-0.58) |
| Delay from ROSC to start of NIRS measurement, hours (95% CI) | 0.94 (0.54-1.32) | 0.03 (0.02-0.05) | 1.01 (0.57-1.41) |
| Duration of NIRS measurement, hours (95% CI) | 17.88 (0.77-72.03) | 0.35 (0.25-0.75) | 59.5 (17.6-73.0) |
| Overall NIRS values, %rSO2 (95% CI) | 64 (50-69) | 50 (39-58) | 67 (60-70) |
| Initial NIRS values post ROSC, %rSO2 (95% CI) | 65 (50-73; min. 30, max. 84) | 50 (40-54; min. 30, max. 57) | 69 (63-75; min. 53, max. 84) |
| NIRS values increasing in first 10 minutes post ROSC, n (%) | 4 (15) | 1 (10) | 3 (18) |
| NIRS values at 24h post ROSC, %rSO2 (95% CI) | 74 (70-76; min. 55, max. 81) | n.a. | n.a. |
| NIRS values at 48h post ROSC, %rSO2 (95% CI) | 70 (67-73; min. 66, max. 75) | n.a. | n.a. |
| NIRS values at 72h post ROSC, %rSO2 (95% CI) | 70 (63-80; min. 62, max. 81) | n.a. | n.a. |

**Supplementary Table S1: Basic demographics of the study population and respective CPR and NIRS characteristics, stratified into the pre- and the in-hospital phase.** CPR = cardiopulmonary resuscitation; NIRS = near-infrared spectroscopy; CPC = cerebral performance category; CA = cardiac arrest; AED = automated external defibrillator; ROSC = return of spontaneous circulation.
