# Supplementary material for: Comprehensive pre- and in-hospital near-infrared-spectroscopy (NIRS) monitoring after return of spontaneous circulation predicts neurological outcome following out-of-hospital cardiac arrest: a prospective observational study and literature review
Source: Front Med (Lausanne). 2025 Aug 15;12:1590908. doi: 10.3389/fmed.2025.1590908 (PMC12394502; doi:10.3389/fmed.2025.1590908)
Supplement: Supplementary file 3 [file Table_2.DOCX]

| **Blood gases and ventilatory settings in post-ROSC care** | | | | |
| --- | --- | --- | --- | --- |
|  | **Total (n=27)** |  |  | **Total (n=27)** |
| **Arterial blood gas analyses** | |  | **Ventilatory settings** | |
| **Initial** | |  | **Initial** | |
| PaO2, mmHg (95% CI) | 106 (86-254) |  | FiO2, % (95% CI) | 50 (33-69) |
| PaCO2, mmHg (95% CI) | 54 (45-66) |  | PEEP, mbar (95% CI) | 6 (6-8) |
| pH, (95% CI) | 7.2 (7.1-7.2) |  | Pmax, mbar (95% CI) | 20 (17-30) |
| Lactate, mmol/L (95% CI) | 7.3 (4.8-13.2) |  | RR, respirations per minute (95% CI) | 9 (7-11) |
| **24h** | |  | **24h** | |
| PaO2, mmHg (95% CI) | 89 (78-107) |  | FiO2, % (95% CI) | 33 (25-50) |
| PaCO2, mmHg (95% CI) | 43 (40-62) |  | PEEP, mbar (95% CI) | 6 (5-7) |
| pH, (95% CI) | 7.4 (7.3-7.4) |  | Pmax, mbar (95% CI) | 20 (15-28) |
| Lactate, mmol/L (95% CI) | 1.0 (0.8-2.2) |  | RR, respirations per minute (95% CI) | 10 (6-13) |
| **48h** | |  | **48h** | |
| PaO2, mmHg (95% CI) | 83 (75-86) |  | FiO2, % (95% CI) | 30 (23-53) |
| PaCO2, mmHg (95% CI) | 43 (37-48) |  | PEEP, mbar (95% CI) | 6 (5-7) |
| pH, (95% CI) | 7.4 (7.4-7.5) |  | Pmax, mbar (95% CI) | 19 (14-25) |
| Lactate, mmol/L (95% CI) | 1.2 (1.1-1.5) |  | RR, respirations per minute (95% CI) | 10 (7-12) |
| **72h** | |  | **72h** | |
| PaO2, mmHg (95% CI) | 85 (76-103) |  | FiO2, % (95% CI) | 39 (21-40) |
| PaCO2, mmHg (95% CI) | 47 (39-48) |  | PEEP, mbar (95% CI) | 5 (5-7) |
| pH, (95% CI) | 7.4 (7.4-7.5) |  | Pmax, mbar (95% CI) | 18 (15-24) |
| Lactate, mmol/L (95% CI) | 0.9 (0.7-1.9) |  | RR, respirations per minute (95% CI) | 12 (8-14) |

**Supplementary Table S2: Blood gases and ventilatory settings in post-ROSC care.** ROSC = return of spontaneous circulation; Pa = partial pressure; O2 = oxygen; CO2 = carbon dioxide; FiO2 = fraction of inspired oxygen; PEEP = positive end-expiratory pressure; Pmax = maximum pressure; RR = respiration rate.
