# Supplementary material for: Comprehensive pre- and in-hospital near-infrared-spectroscopy (NIRS) monitoring after return of spontaneous circulation predicts neurological outcome following out-of-hospital cardiac arrest: a prospective observational study and literature review
Source: Front Med (Lausanne). 2025 Aug 15;12:1590908. doi: 10.3389/fmed.2025.1590908 (PMC12394502; doi:10.3389/fmed.2025.1590908)
Supplement: Supplementary file 4 [file Table_3.DOCX]

|  | **CPC 3 or 4 (n=3)** | **CPC 5 (n=17)** | **p-value** |
| --- | --- | --- | --- |
| rSO2, % (IQR) | 65 (62-68) | 64 (47-66) | 0.491 |

**Supplementary Table S3:** Comparison of regional cerebral oxygen saturation (rSO2) between patients with a cerebral performance category (CPC) at hospital discharge of 3 or 4 vs. those with a CPC of 5 (deceased) via Mann-Whitney-U test.
